# The HEAL-HOA Dual Randomized Controlled Trial: 12-month Effects on Loneliness in Older Adults

**DOI:** 10.1093/geroni/igaf122.3123

**Published:** 2025-12-31

**Authors:** Vivien Tang, Da Jiang, Jojo Yan Yan Kwok, Namkee Choi, Dannii Yeung, Lisa Warner, Rainbow Tin Hung Ho, Kee-Lee Chou

**Affiliations:** The Education University of Hong Kong, Hong Kong, China (People’s Republic); The Education University of Hong Kong, Hong Kong, China (People’s Republic); The University of Hong Kong, Hong Kong, China (People’s Republic); The University of Texas at Austin, Austin, Texas, United States; City University of Hong Kong, Hong Kong, China (People’s Republic); MSB Medical School Berlin, Berlin, Berlin, Germany; The University of Hong Kong, Hong Kong, China (People’s Republic); The Education University of Hong Kong, Hong Kong, China (People’s Republic)

## Abstract

Loneliness is a growing public health concern, yet the long-term effectiveness of psychosocial interventions remains uncertain. As part of the Helping Alleviate Loneliness in Hong Kong Older Adults, a dual randomized controlled trial conducted during the COVID-19 pandemic, this study focused on the long-term outcomes of older adults (Mage=76.6, SDage=7.8) who lived alone, felt lonely, faced financial difficulties, and were digitally excluded. The interventions consisted of behavioral activation (Tele-BA), mindfulness (Tele-MF), and befriending (Tele-BF: attention control). Each intervention comprised eight 30-minute telephone-delivered sessions over one month, led by trained laypersons. The lay interventionists were older adult peers who also experienced loneliness. Outcomes were assessed at baseline and 1-, 3-, 6-, and 12-month post-intervention. Compared to Tele-BF, Tele-BA and Tele-MF showed sustained reduction of loneliness at12-month follow-ups. Secondary outcome were also found to improved significantly for sleep quality, life satisfaction, and psychological well-being. Social isolation at 6 months mediated the effects of Tele-BA and Tele-MF at the 12-month follow-up, with a larger increase in social network leading to a more substantial reduction in loneliness. This RCT demonstrated the feasibility of scalable telephone-based interventions beyond the pandemic, with Tele-BA emerging as a promising intervention for alleviating loneliness and improving overall well-being in older adults. Future psychosocial interventions for late-life loneliness should specifically target social isolation, as it mediates the long-term effects of Tele-BA and Tele-MF on loneliness.

